# Wound Healing and Acute Dermal Toxicity Studies of *Ludwigia octovalvis* (Jacq.) P. H. Raven (Onagraceae) in *Sprague–Dawley* Rats

**DOI:** 10.1155/2024/9576349

**Published:** 2024-09-19

**Authors:** Martin Boadi, Stephen Yao Gbedema, Yaw Duah Boakye, Marcel Tunkumgnen Bayor, Eugene Agyei Aboagye

**Affiliations:** ^1^ Department of Pharmaceutical Microbiology School of Pharmacy University of Health and Allied Sciences, Ho, Ghana; ^2^ Department of Pharmaceutics Faculty of Pharmacy and Pharmaceutical Sciences College of Health Sciences Kwame Nkrumah University of Science and Technology, Kumasi, Ghana; ^3^ Department of Pathology Manhyia District Hospital, Ashanti Region, Kumasi, Ghana

## Abstract

**Introduction:**

The aerial part of *Ludwigia octovalvis* has been used traditionally in some parts of Asia for the management of wounds owing to the presence of phytochemicals such as tannins, flavonoids, and triterpenoids among others. The incidence of wounds, their associated complications, and the cost of wound care are on the increase globally, therefore, the need to develop alternative wound care agents. The aim of this study was to scientifically investigate the wound healing potential of the ethanolic extract of *L. octovalvis* using the excision wound healing model in rats and also carry out an acute dermal toxicity investigation of the plant extract.

**Method:**

A 70% ethanol extract of *L. octovalvis* was prepared for the wound healing activity using the excision wound healing model in *Sprague–Dawley* rats. Aqueous creams (1, 3, and 10%) were prepared and topically applied to the wounds once daily according to the groups of animals. The wounds were assessed for rates of wound closure on days 3, 5, 7, 9, and 11. Re-epithelialization periods were also determined. Sections of wound tissues obtained on day 13 were subjected to histological investigations. An acute dermal toxicity of the plant extract was investigated.

**Results:**

*L. octovalvis* treatment (1, 3, and 10%) exhibited a mean percentage wound contraction range of 85.36 ± 7.22–94.14 ± 2.23 on day 11. The extract exhibited re-epithelialization periods of 17.3 ± 1.2, 19.8 ± 2.6, and 16.0 ± 1.7 days for the 1, 3, and 10% extract creams, respectively, whereas the cream-only and 1% silver sulfadiazine treatments resulted in a re-epithelialization period of greater than 28 days. Histopathological investigation revealed enhanced fibroblast infiltration and collagen deposition in the treatment groups. No adverse reaction was observed in the acute dermal toxicity study.

**Conclusions:**

Extract of *L. octovalvis* exhibited wound healing by enhancing wound contraction, re-epithelialization, fibroblast infiltration, and collagen deposition at the wound site. The extract did not exhibit any toxic reaction in the acute dermal toxicity study.

## 1. Introduction

Wounds result from physical, chemical, thermal, microbial, and immunological injury to parts of the body to bring about the breakdown of continuity of function of the affected body tissues. Types of wounds include incision wounds, aberrations, contusions, lacerations, ulcers, burns, pressure, and diabetic wounds [1]. Four overlapping phases are involved in wound healing to restore the structural and functional continuity of damaged tissues. These phases are haemostasis, inflammation, proliferation, and remodeling [2]. The various phases of wound healing employ the integration of both cellular and biochemical processes. Growth factors such as hypoxia-inducible growth factor (HGF), platelet-derived growth factor (PDGF), epithermal growth factor (EGF), and transforming growth factor *β* (TGF-*β*) initiate and promote the wound healing process [3]. Macrophages, endothelial cells, fibroblasts, and epidermal cells also play vital roles in bringing about complete healing. The actions of these molecular and cellular agents are precise, but their interruption can lead to improper healing, delayed healing, or chronic wounds [4]. When an acute wound is not managed well, it can also result in chronic wounds.

Wound healing is characterized by parameters such as wound contraction, re-epithelialization, collagen deposition, and granulation tissue formation. Wound contraction is a centripetal movement of the edges of a full-thickness wound to ensure closure of the wound [5]. This process is an indication of successful granulation tissue formation, angiogenesis, fibroblasts, and keratinocytes infiltration to the wound bed and re-epithelialization [6]. Re-epithelialization is a crucial step in the healing process and it is used to identify effective healing. In the absence of re-epithelialization, a wound cannot be considered healed [7]. The process of coating the skin's epithelium is known as epithelialization. It entails the migration and multiplication of keratinocytes and other epithelial cells throughout the wound bed [8]. Proliferated fibroblasts in the wound matrix release matrix proteins particularly collagen, which maintain the strength and integrity of the wound tissues. They play a key role in the proliferative and remodeling phases of wound healing [7]. Primarily, collagen acts as a base for the intracellular matrix formation within the wound. Both type I and III collagens are produced in the wound with type III being synthesized in higher proportion at the initial stages [9]. Angiogenesis, a key component of the extracellular matrix restores blood capillaries in the wounds. To maintain tissue viability, new blood vessel establishment is critical at all phases of the wound healing process [7]. The establishment of all components of healing brings about a successfully healed wound.

The occurrence of wounds and their associated complications are on the increase globally. A report in 2019 stated that chronic nonhealing wounds affected 10.5 million people (out of the numerous wound patients) in the United States alone. In recent times, chronic wounds arising from varying etiologies affect both adult and younger populations. There has also been a rise in the cost associated with wound care worldwide. The global wound care market for 2022 was valued at $20.8 million and it is estimated to reach $30.52 million in the year 3030 [10].

Agents such as antibiotics, analgesics, and nonsteroidal anti-inflammatory drugs (NSAIDs) have been employed in wound management. The numerous side effects posed by these agents along with the rising cost of wound care have necessitated the search for new, effective, and less toxic agents as alternatives for the management of wounds. The folkloric uses of plants in the management of wounds have led to the employment of phytochemicals such as triterpenes, flavonoids, and alkaloids in wound healing [11]. These agents pose fewer side effects and have the potential to influence one or two phases of the wound-healing process.

**Figure 1:**
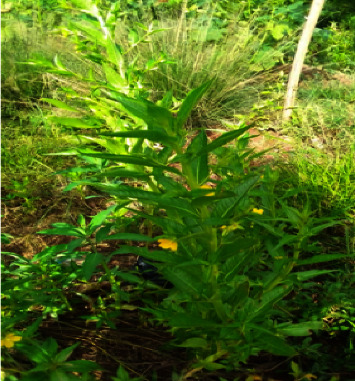
Aerial parts of the *L. octovalvis* plant.

*Ludwigia octovalvis* (primrose willow) (Figure 1) is a medicinal plant belonging to the division Angiospermatophyta, class Dicotyledonae, order Myrtales, and family Onagraceae. It is an erect, branched, perennial flowering shrub that grows at the borderline of water bodies, swampy areas, and other flooded tropical environments [12]. In some parts of Asia, the plant is traditionally consumed as a drink for treating and managing various conditions including oedema, nephritis, hypotension, and diabetes. It is also used in the treatment of wounds and skin diseases [13–15]. In Mexico, a decoction of the whole plant is used to manage chickenpox, diarrhea, dysentery, cough, fever, headache, skin diseases, and fungal infection of the toes.

The ethanolic extract of the leaves of *L. octovalvis* has shown antimicrobial activity against *E. coli*, *P. aeruginosa,* and *S. aureus* [15]. Nanda et al. [16] reported the antibacterial activity of the whole plant against *S. aureus, S. pyogenes, E. faecalis, Propionibacterium acnes, K. pneumonia, P. aeruginosa* and fungi such *C. albicans, C. tropicalis, C. krusei, Cryptococcus marinus, Microsporum gypseum, Trichophyton rubrum*, *Epidermophyton floccosum*, and *Aspergillus niger*. Research has also reported on the presence of *α*-glucosidase inhibitors and antioxidant and antiaging potential of the plant [14, 17]. The antimicrobial potential and folkloric use of *L. octovalvis* in some parts of the world for wound management make it suitable for research into its wound healing and dermal toxicity potential. The aim of this study was to investigate the influence of 70% ethanolic extract of *L. octovalvis* on excision wound healing and also its acute dermal toxicity profile in rats.

## 2. Materials and Methods

### 2.1. Collection of Plant Material

The shoot system of the plant was collected from Kyebi (EE-0017-6634) in June 2020. It was authenticated in the KNUST department of herbal medicine and the voucher specimen (KNUST/HM1/2022/WP002) was deposited in the herbarium of the Faculty of Pharmacy and Pharmaceutical Sciences, College of Health Sciences, Kwame Nkrumah University of Science and Technology (KNUST). The collected plant material was shade-dried for three weeks prior to extraction.

## 3. Extraction of Plant Material

The dried plant material was milled into powder using a locally manufactured milling machine. 100 g of the powdered material was cold macerated in 500 mL of 70% ethanol (v/v) for 72 hours after which it was filtered through Whatman No. 1 filter paper. The filtrate was concentrated at 50°C (using a rotary evaporator) to about 100 mL of syrupy extract which was further dried in an oven (Gallenkamp, England) at 50°C for 48 hours. The dried extract (ELO) was kept at room temperature for further studies.

### 3.1. Aqueous Cream Formulation

The extract obtained was formulated into aqueous creams according to the method described in the British Pharmacopoeia (BP, 2007). Three extract concentrations (1, 3, and 10%) were formulated and kept at room temperature. In the formulation of the creams, a total of 1 kg of the emulsifying ointment was first prepared using the formula in Table 1.

These ingredients (Table 1) were weighed and heated together to melt completely in a stainless steel container. After thorough mixing, the setup was allowed to cool and solidify. The emulsifying ointment was used to prepare 100 g of each of the various aqueous creams using the formula in Table 2.

Appropriate amounts of the ingredients described in Table 2 were weighed into stainless steel containers. The mixture for each extract concentration was heated on a water bath at 80°C and then mixed together using a homogenizer (Janke & Kunkel GmbH, UK) at 15,000 rpm until a homogenous mixture was obtained. The molten products were allowed to cool and solidify. They were kept in glass containers, covered tightly, and then kept at room temperature until needed.

### 3.2. Experimental Animals

Thirty-six *Sprague*–*Dawley* rats weighing 184–207 g were purchased from the Noguchi Memorial Institute for Medical Research, University of Ghana, Legon, Ghana, and transported to the animal house of the department of pharmacology, Faculty of Pharmacy and Pharmaceutical Sciences, College of Health Sciences, KNUST, Kumasi, Ghana where the wound healing experiment was performed. It was ensured that the animals had not been previously subjected to any treatment. The animals were randomly grouped into six with each group containing 6 members of equal sexes. One animal within each group was separately kept in a clean aluminum cage maintained at room temperature (28–30°C) with a relative humidity of 60–70% and exposed to 12 hours of light-dark cycles. They were fed twice a day with equal quantities of commercial rodent feed (GAFCO, Ghana) and provided with clean water. Institutional ethical clearance (KNUST 0037) was obtained from the Animal Research Ethics Committee (AREC) of KNUST prior to the wound healing activity.

### 3.3. Excision Wound Healing Study

The experimental animals were acclimatized for 7 days after which their dorsal hairs were shaved with razor blades. They were then anesthetized with 120 mg/kg per body weight of ketamine hydrochloride (Pfizer, New York, USA) subcutaneously [4]. The shaved area of each animal was cleaned with 70% ethanol after which an excision wound (area 118–142 mm^2^) was created at the dorsal region of each animal with the help of clean sterile surgical scissors and toothed forceps. In brief, the area to be excised was outlined with a permanent marker. The skin was held with the forceps and raised after which a small excision was made. The wound was finally extended using the outline as a guide [18]. About 0.5 g of the cream was applied topically to the wounds once daily according to the treatment plan in Table 3. All wounds were cleaned daily with normal saline prior to the application of the cream.

### 3.4. Determination of the Percentage Rate of Wound Contraction

The wound areas were measured on days 1, 3, 5, 7, 9, and 11 using the graphical method [19]. In brief, the perimeter of each wound was traced on a transparent plastic material with a permanent marker. They were then evaluated on a 1 mm^2^ scale graph sheet. The measured wound areas were used to determine the percentage of wound contraction by using the following formula:

(1) $$\begin{array}{c} \text{percentage wound contraction}=\frac{A_{0}-A_{t}}{A_{0}}\times 100\%, \end{array}$$

where *A*_0_ is the initial wound area and *A*_*t*_ is the area of the wound on a specific day [20]. A graph of percentage wound contraction against time was plotted for days 3, 5, 7, 9, and 11. One-way ANOVA followed by Bonferroni's post hoc analysis was used to evaluate the significant difference (*p* < 0.05) between the treatment groups and the controls on days 3, 5, 7, 9, and 11.

### 3.5. Assessment of the Re-epithelialization Period

The wounds were monitored for up to 28 days for complete re-epithelialization, noting the day the Escher separates itself and falls from the wound surface leaving behind no residual raw wound [21]. The animal whose Escher did not separate within the 28 days was noted as having a re-epithelialization period greater than 28 days. One-way ANOVA followed by Bonferroni's post hoc analysis was used to determine the significant difference between the treatment groups and the controls.

### 3.6. Histological Assessment of Wound Tissues

Wound tissues obtained on day 13 postwounding were processed for histological studies using standard protocols. In brief, wound tissues were fixed in a 10% neutral buffered formalin (NBF) solution for 24 hours. They were then washed in phosphate-buffered saline (PBS) to remove the NBF. The tissues were dehydrated in increasing concentrations of ethanol (30, 40, 50, 60, 70, 80, 90, 95, 98% (v/v)). The ethanol was removed by dipping the tissues in xylene and subsequently embedding them in paraffin wax. They were allowed to solidify to form blocks [22]. The tissue blocks were sectioned into 5 *μ*m using a microtome device (Leica RM2125 RTS, Leica Biosystems, USA). The sections were then mounted on glass slides (75 × 25 mm) and deparaffinized in xylene. The deparaffinized sections were hydrated and stained with hematoxylin and eosin (H&E) stains. The stained sections were viewed and analyzed under a microscope (Leica DM500, Leica Microsystems (Schweiz) AG, Switzerland). The presence of wound healing indicators such as macrophages, fibroblasts, lymphocytes, neutrophils, blood vessels, and collagen deposition among others in the wound tissues was evaluated. The levels of these observable wound healing indicators were scored and recorded as low (+), moderate (++), and high (+++). Representative micrographs of stained sections for each slide were also taken with a BMS microscope camera (BMS, Netherlands).

The procedure was repeated for the Van Gieson stain to stain collagen fibers. Ten micrographs were randomly obtained from different fields of the stained sections of each treated group. The micrographs were then processed in ImageJ software to estimate the percentage area of the sections that stained positive for collagen fibers. A graph of the total area that stained positive for collagen was plotted. One-way ANOVA followed by Bonferroni's post hoc analysis was used to compare the treatment groups and the controls.

### 3.7. Acute Dermal Toxicity Studies

The *L. octovalvis* extract was subjected to a dermal toxicity study. Nine female *Sprague–Dawley* rats (189–206 g) were purchased from the Noguchi Memorial Institute for Medical Research (Legon) and transported to the animal house of the department of pharmacology, KNUST. They were randomly grouped into three with each group consisting of 3 members. They were kept at room temperature (28–30°C) with 12-hour light-dark cycles. They were provided with a standard rodent pellet diet (GAFCO, Ghana) and water *ad libitum* throughout the study. The rats were acclimatized for 7 days after which their dorsal lumbar region furs were shaved with a razor blade [23]. The weights of the animals were determined and recorded on days 1 (before the application of the test agents), 7, and 14.

The acute dermal toxicity study was conducted in accordance with the Organization for Economic Co-operation and Development (OECD) guidelines for the Testing of Chemicals, Test guideline 404 (2015) and the Fixed Dose Procedure guideline 402 (2017). In brief, a fixed dose of 2000 mg/kg body weight of the extract was reconstituted in distilled water. A 0.5 mL portion of the reconstituted extract was applied to a gauze patch (2 × 2 cm). The patch was applied onto the shaved skin of the rats of group 1 (Table 4). The patches were then held in close contact with the skin by means of a nonirritating adhesive tape for 24 hours after which it was removed. The exposed areas were cleaned with sterile distilled water and were observed for signs of oedema and erythema at 1, 24, 48, and 72 hours postpatch removal. Observations were further made on days 7 and 14 for signs of oedema, erythema, irritation, corrosion, and also changes in body weight. Prior to the removal of the patches, the animals were also observed for signs of changes in fur, eye, respiratory, and general behavior patterns. They were also observed for the presence of tremors, convulsions, salivation, diarrhea, sleep, and coma (OECD 402, 2017).

## 4. Results

### 4.1. Percentage Rates of Wound Contraction

**Figure 2:**
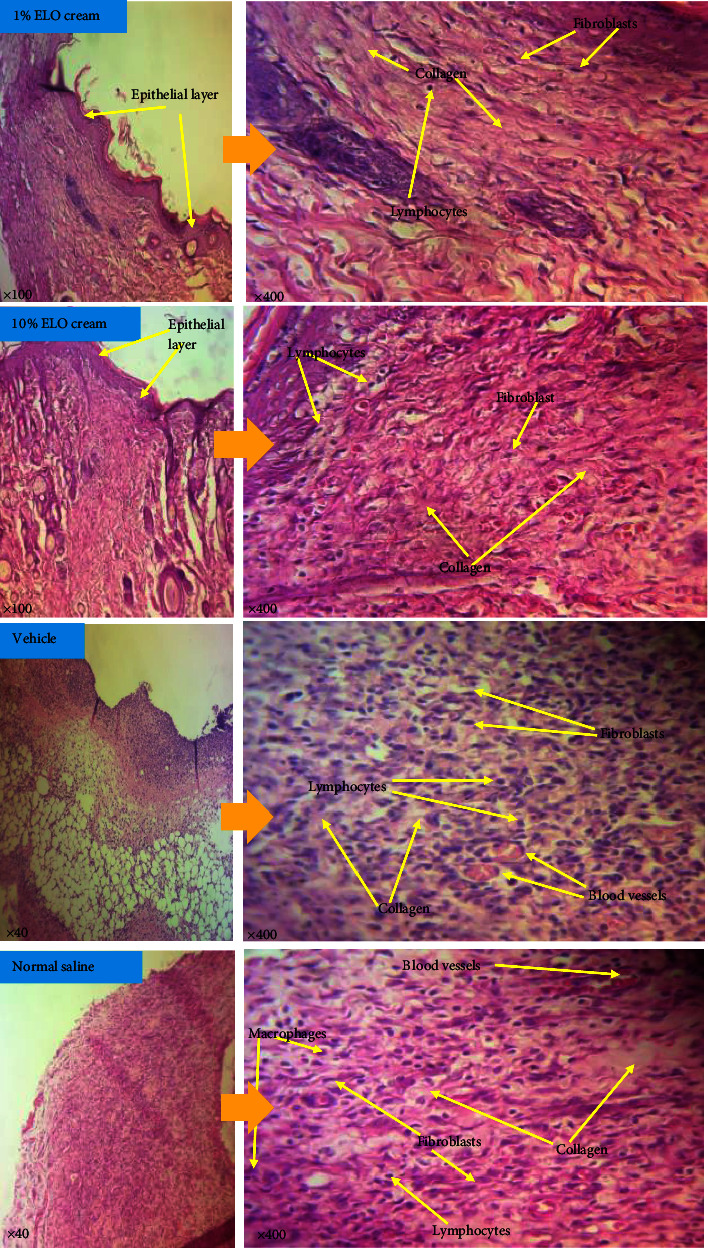
Micrographs of the H&E stained wound tissues treated with 70% ethanol extract of *L. octovalvis* (1% and 10%) and the controls (vehicle and normal saline)-treated wound.

**Figure 3:**
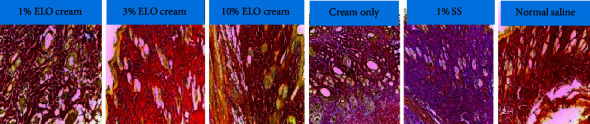
Micrographs of the collagen-stained wound tissues. Pink or purple sections denote collagen deposited area. 1% SS: 1% silver sulfadiazine.

The rates of wound contraction for animals treated with the extract (1, 3, and 10%) are illustrated in Figure 2. A sharp reduction in wound area was observed in the groups treated with creams containing 3 and 10% of the extract between days 5 and 7 and proceeded gradually to day 11. There was a significant difference (*p* < 0.05) in the percentage rates of contraction between the 3% extract-treated group and the vehicle-treated group on day 7. In the case of the 1% extract cream-treated group, there was an increased wound contraction between days 5 and 9 followed by a gradual rate of contraction between days 9 and 11. There was a significant difference (*p* < 0.05) in the rate of wound contraction between the 1% extract-treated group and the vehicle-treated group on day 9.  Figure 3 shows a reduction in wound sizes in the various treatment groups.

### 4.2. Assessment of the Re-Epithelialization Period

The animals treated with the 10% extract cream had the lowest re-epithelialization period of 16.0 ± 1.7 days followed by those treated with 1% extract cream with a mean re-epithelialization period of 17.3 ± 1.2 days. The vehicle- and 1% silver sulfadiazine-treated groups showed re-epithelialization periods greater than 28 days (Table 5).

### 4.3. Histological Evaluation

**Figure 4:**
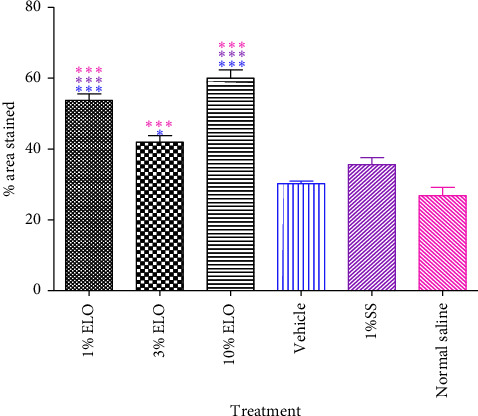
Mean percentage area stained with the Van Gieson stain in the extract-treated groups compared to the controls. ^∗∗∗^Significant at *p* < 0.001. ^∗^Significant at *p* < 0.05. 1% SS: 1% silver sulfadiazine.

The histological evaluation aimed at assessing and identifying cells involved in wound healing. Sections of wound tissues were stained with hematoxylin and eosin (H&E) to enable differentiation between nuclear bodies and other wound tissue components such as collagen in the extracellular matrix. Indicators such as fibroblasts, macrophages, neutrophils, lymphocytes, blood vessels, collagen fibers, and epithelial layer formation were assessed in this study. A representative image (Figure 4) for each treatment group was taken and observations made were recorded and tabulated (Table 6).

### 4.4. Collagen Staining

**Figure 5:**
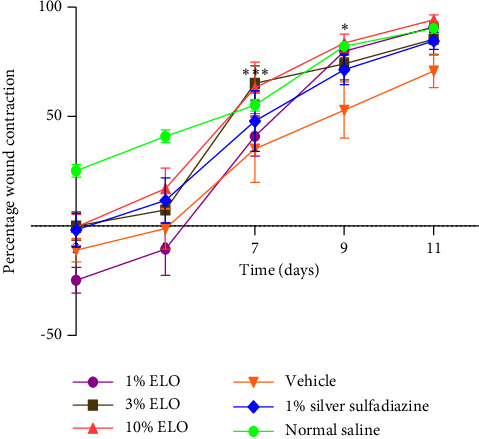
Rates of contraction of wounds treated with *L. octovalvis* creams. Note: one-way ANOVA followed by Bonferroni's post hoc analysis was used for comparison between the different groups. ^∗∗∗^Significant difference (*p* < 0.05) between the 3% ELO-treated and the vehicle-treated groups. ^∗^Significant difference (*p* < 0.05) between the 10% ELO-treated and the vehicle-treated groups.

**Figure 6:**
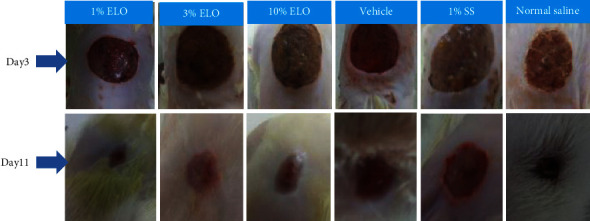
Areas of wounds treated with ELO creams (1%, 3%, and 10%), cream only, 1% silver sulfadiazine, and normal saline on days 3 and 11.

The Van Gieson staining of collagen fibers (Figure 5) revealed that the 10% extract-treated group exhibited a higher collagen deposition as compared to the other treatment groups. Statistically, there were significant differences (*p* < 0.001) between the amount of collagen deposited in both the 1% and 10% extract-treated groups compared to all the controls. A significant difference existed between the 3% treated group and both the vehicle- (*p* < 0.05) and the normal saline (*p* < 0.01)-treated groups (Figure 6).

### 4.5. Acute Dermal Toxicity Study of the Extract

Evaluation of the effect of topical application of *L. octovalvis* extract revealed no observable signs of oedema or erythema in the treated groups. The 5% NaOH treatment resulted in oedema, erythema, and finally corrosion. There was no incidence of fatality and a significant decrease in body weight in all treated groups during the study period (Table 7). There were also no changes in fur, eye, respiratory, and behavior patterns during the study period. Also, there were no signs of tremors, convulsions, salivation, diarrhea, sleep, and coma.

## 5. Discussion

Wound healing is a complex process involving overlapping biochemical and cellular mechanisms to restore damaged tissues [24, 25]. There are four main overlapping processes involved in wound healing. These are haemostasis, inflammation, proliferation, and remodeling. Haemostasis is activated when platelets come into contact with exposed collagen at the site of injury resulting in platelet aggregation and the release of clotting factors. The clotting factors bring about the deposition of fibrin clot at the site of injury. The fibrin clot serves as a provisional matrix for invading wound healing cells and sets the stage for the subsequent events of healing [26]. The clot also seals off the wound surface against the entry of microbes. The inflammatory phase is characterized by infiltration of neutrophils, macrophages, and lymphocytes. Microbes and cellular debris are cleared from the wound tissues by the inflammatory cells. Macrophages are key players in wound healing and their activities control most events in the healing process [27]. The proliferative phase is also characterized by granulation tissue formation. In this phase, there is infiltration of fibroblasts, endothelial cells, and epithelial cells at the wound site. Fibroblasts are responsible for the deposition of collagen into the extracellular matrix. Myofibroblasts differentiate from fibroblasts and are responsible for wound contraction. Endothelial cells are responsible for blood vessel regeneration whereas epithelial cells bring about re-epithelialization of the wound [27, 28]. A series of events in the proliferative phase bring the skin to its physiological function. The remodeling phase lasts for years. In this phase, all processes that started in the previous phases come to a halt. Macrophages, myofibroblasts, and endothelial cells go into apoptosis leaving collagen and extracellular matrix protein-rich tissues. There is a continuous regulation of the skin's integrity and homeostasis in the remodeling phase of the healing process [29].

All the phases of wound healing can be promoted or enhanced by wound healing agents such as phytochemicals [30]. In this study, the wound healing potential of aqueous creams (1, 3, and 10%) containing 70% (v/v) ethanol extract of *L. octovalvis* was assessed using the excision wound healing model. The rate of healing was evaluated by determining the rates of wound closure and re-epithelialization periods. Wound tissues were also evaluated for the presence of macrophages, neutrophils, lymphocytes, blood vessels, and collagen deposition among others.

Wound contraction is the centripetal movement of the edges of a full-thickness wound to ensure the closure of the wound [31, 32]. This process is an indication of successful granulation tissue formation, angiogenesis, and fibroblasts and keratinocytes infiltration to the wound bed [1]. Treatment of wounds with an aqueous cream containing *L. octovalvis* extract (10%) showed enhanced wound closure. On day 11 postwounding, the extract (10%) exhibited a percentage rate of wound closure of 94.14 ± 2.27% (Figure 2). The observed rate of wound closure of the extract was comparable to those of silver sulfadiazine and the normal saline-treated groups. The enhanced rate of wound closure of the 10% extract-treated group compared to the controls is an indication of a wound healing potential.

An essential component of wound healing that is used as a defining factor of successful healing is re-epithelialization. A wound cannot be considered healed in the absence of an epithelial layer [2]. The process of re-epithelialization involves the proliferation and migration of epithelial cells such as keratinocytes across the wound bed [33]. The observed re-epithelialization periods were 17.3 ± 1.2, 19.8 ± 2.6, and 16.0 ± 1.7 days for the 1, 3, and 10% extract-treated groups, respectively. These observations agree with the observed rates of wound closure in this study. Thus, wounds with an enhanced rate of closure resulted in a shorter re-epithelialization period. The histological evaluation (Table 6) also revealed the presence of an epithelial layer in the treatment groups with relatively higher rates of wound closure to indicate the extent of healing. The re-epithelialization periods for the extract-treated groups were promoted compared to the vehicle- (>28), silver sulfadiazine- (>28 days), and normal saline (24.0 ± 1.4 days)-treated groups.

The wound tissues of the extract-treated groups exhibited moderate to high infiltration of fibroblasts. This suggests that *L. octovalvis* influenced wound healing by enhancing the migration or proliferation of fibroblasts to the wound site [34]. In wound tissues, fibroblasts produce collagen, elastin, and glycosaminoglycans. These extracellular matrix components promote cellular migration and interaction within the wound matrix [35]. Collagen forms the main constituent of the extracellular matrix and it is responsible for conveying tensile strength to the scar [36, 37]. In the histological evaluation, moderate to high quantities of collagen were deposited in the various treatment groups. The amounts of collagen deposited in the wound tissues were analyzed by staining sections of the tissues with the Van Gieson stain. A significant (*p* < 0.001) amount of collagen was deposited in the 1% and 10% extract-treated wound tissues compared to the controls. High cross-linking of collagen fibers was observed in the wound tissues treated with the extract (Figure 6). The high level of collagen deposited is an evidence of the activities of proliferated fibroblasts in the proliferative phase of the healing process [6, 37, 38].

Angiogenesis re-establishes blood supply to wounded tissues. It is stimulated by hypoxic conditions that result from reduced blood supply and accelerated metabolism of cells at sites of injury [39]. Histological evaluation of the wound tissues (Table 6) revealed moderate levels of vascularization in the extract-treated groups and hence promoted the healing process [40]. This observation is in agreement with that reported by [41] where *Curcuma purpurascens* promoted wound healing by enhancing angiogenesis.

Agents that are able to stimulate re-epithelialization, fibroblasts and endothelial cells proliferation and migration, and also increase collagen synthesis are considered as wound healing agents. If two or more of these biological activities are promoted by an agent, the agent is described as a good wound healing agent [6, 42]. The enhanced rate of wound contraction, re-epithelialization periods, and significant deposition of collagen fibers observed in the *L. octovalvis* extract treatment indicate that it possesses wound healing potential.

In the acute dermal toxicity study, all rats administered with the 2000 mg/kg single dose of the extract revealed no changes in fur, eye, respiratory, and behavior patterns during the study period. Also, there were no signs of tremors, convulsions, salivation, diarrhea, sleep, and coma. Irritation and corrosion were also not observed. No case of fatality was observed. The rats rather gained weight (Table 7). This observation agrees with that reported by the authors in [43] where rats administered with the ethanolic extract of *Morinda citrifolia* showed no toxic effect. A report by the authors in [44] also revealed no toxic effect in the dermal application of the *Plumbago zeylanica* extract on rats. The absence of toxic effects in this current study shows that the short-term dermal application of aqueous cream containing the extract of *L. octovalvis* in the treatment and management of wounds may not cause adverse effects [45]. The observations made in this study justify the folkloric use of *L. octovalvis* in wound healing. Ethanol (70% v/v) extract of the plant, therefore, can be employed in herbal wound care formulations.

## 6. Conclusion

Extract of *Ludwigia octovalvis* possesses wound healing potential by enhancing wound contraction, re-epithelialization, and collagen synthesis. In addition, no toxic effect was observed in the acute dermal toxicity study and, therefore, can be useful for topical application of wounds to ensure successful healing.

## Figures and Tables

**Table 1:** Formula used for emulsifying ointment preparation.

Ingredients	Quantity (g)
Emulsifying wax	300
White soft paraffin	500
Liquid paraffin	200

**Table 2:** Formula for *L. octovalvis* extract's cream formulation.

Ingredient	Creams concentration/amounts (g)		
	1	3	10
Emulsifying ointment	29.7	29.1	27.0
Extract	1.0	3.0	10.0
Distilled water	69.3	67.9	63.0

**Table 3:** Plan for wound treatment.

Groups (*n* , 6)	Treatment
1	1% extract cream
2	3% extract cream
3	10% extract cream
4	Aqueous cream (vehicle)
5	1% Silver sulfadiazine
6	Normal saline treatment

**Table 4:** Histological evaluations of wound tissues.

Treatment	Histological parameters							Remarks
	Mac	Neu	Lym	Fib	BV	Collagen	EL	
1% ELO cream	−	−	+	++	++	++	Present	Healing
3% ELO cream	−	−	+	++	+	++	Absent	Healing
10% ELO cream	−	−	+	+++	++	+++	Present	Healed
Vehicle	−	−	++	+	++	++	Absent	Healing
1% silver sulfadiazine	−	−	−	+	+	+	Absent	Healing
Normal saline	+	−	−	+	++	++	Absent	Healing

Mac, macrophages; Neu, neutrophils; Lym, lymphocytes; Fib, fibroblasts; BV, blood vessels; EL, epithelial layer; −, absent; +, low; ++, moderate; +++, high.

**Table 5:** Plan for acute dermal toxicity study.

Group (*n* , 3)	Treatments
1	Extract
2	5% NaOH
3	Untreated

**Table 6:** Re-epithelialization periods of the wound for various treatments.

Group	Treatment	Re-epithelialization period
1	1% ELO extract	17.3 ± 1.2^a,b^
2	3% ELO extract	18.8 ± 2.6^a,b^
3	10% ELO extract	16.0 ± 1.7^a,b,c^
4	Vehicle	>28
5	1% silver sulfadiazine	>28
6	Normal saline	24.0 ± 1.4

Values are expressed as mean ± SEM. ^a^Significant difference (*p* < 0.05) existed between the treatment group and the cream-only treated group. ^b^Significant difference (*p* < 0.05) existed between the treatment group and the 1% silver sulfadiazine-treated group. ^c^Significant difference (*p* < 0.05) existed between the treatment group and the untreated group.

**Table 7:** Acute dermal toxicity study of the *L. octovalvis* extract.

Treatment	Group/members	Changes in weight/g			Changes in skin		Remarks
		Day 0	Day 7	Day 14	Oedema	Erythema	
Extract	1	173.3	163.2	162.3	Absent	Absent	No side effect
	2	134.6	136.3	139.0	Absent	Absent	No side effect
	3	162.4	160.9	165.4	Absent	Absent	No side effect

5% NaOH	1	165.3	166.0	170.3	Present	Present	Corrosive
	2	129.3	133.5	135.3	Present	Present	Corrosive
	3	129.9	136.2	138.6	Present	Present	Corrosive

Untreated	1	182.6	184.4	192.2	Absent	Absent	No side effect
	2	164.5	172.5	187.3	Absent	Absent	No side effect
	3	114.2	134.5	145.8	Absent	Absent	No side effect

## Data Availability

The data used to support the findings of this study are available from the corresponding author upon request.

## References

[1] Somboonwong J., Kankaisre M., Tantisira B., Tantisira M. H. (2012). Wound healing activities of different extracts of *Centella asiatica* in incision and burn wound models: an experimental animal study. *BMC Complementary and Alternative Medicine*.

[2] Juszczak A. M., Jakimiuk K., Czarnomysy R. (2022). Wound healing properties of *Jasione montana* extracts and their main secondary metabolites. *Frontiers in Pharmacology*.

[3] Nagar H. K., Srivastava A. K., Srivastava R., Kurmi M. L., Chandel H. S., Ranawat M. S. (2016). Pharmacological investigation of the wound healing activity of *Cestrum nocturnum* (L.) ointment in wistar albino rats. *Journal of Pharmaceutics*.

[4] Agyare C., Owusu-ansah A., Poku P., Ossei S., Apenteng J. A., Boakye Y. D. (2014). Wound healing and anti-infective properties of *Myrianthus arboreus* and alchornea cordifolia. *Medicinal Chemistry*.

[5] Tang T., Yin L., Yang J., Shan G. (2007). Emodin, an anthraquinone derivative from *Rheum officinale* Baill, enhances cutaneous wound healing in rats. *European Journal of Pharmacology*.

[6] Boakye Y. D., Agyare C., Ayande G. P. (2018). Assessment of wound-healing properties of medicinal plants: the case of *Phyllanthus muellerianus*. *Frontiers in Pharmacology*.

[7] Pastar I., Stojadinovic O., Yin N. C. (2014). Epithelialization in wound healing: a comprehensive review. *Advances in Wound Care*.

[8] Esimone C. O., Nworu C. S., Jackson C. L. (2008). Cutaneous wound healing activity of a herbal ointment containing the leaf extract of *Jatropha Curcas* L. (Euphorbiaceae). *International Journal of Applied Research in Natural Products*.

[9] Demling R. H. (2009). Nutrition, anabolism, and the wound healing process: an overview. *Eplasty*.

[10] Sen C. K. (2023). Human wound and its burden: updated 2022 compendium of estimates. *Advances in Wound Care*.

[11] Kuluvar G., Mahmood R., Ahamed B. M. K., Babu P. S., Krishna V. (2009). Wound-healing activity of *Clerodendrum infortunatum* L. Root extracts. *International Journal of Biomedical and Pharmaceutical Sciences*.

[12] Ribeiro J. P. N., Matsumoto R. S., Lima M. I. S. (2012). The effects of seasonal change of water level in an estuary on *Ludwigia octovalvis* (Jacq.) P. H. Raven (Onograceae) growth. *Acta Botanica Brasilica*.

[13] Ramírez G., Zavala M., Pérez J., Zamilpa A. (2012). In vitro screening of medicinal plants used in Mexico as antidiabetics with glucosidase and lipase inhibitory activities. *Evidence-Based Complementary and Alternative Medicine*.

[14] Yakob H. K., Sulaiman S. F., Uyub A. M. (2012). Antioxidant and antibacterial activity of *Ludwigia octovalvis* on *Escherichia coli* O157:H7 and some pathogenic bacteria. *World Applied Sciences Journal*.

[15] Lin W. S., Lo J. H., Yang J. H. (2017). *Ludwigia octovalvis* extract improves glycemic control and memory performance in diabetic mice. *Journal of Ethnopharmacology*.

[16] Nanda U. N., Barik B. B., Sahoo S. (2008). Antimicrobial activity of *Ludwigia octovalvis* (JACQ) Raven against selected human dermatological pathogens. *Indian Drugs*.

[17] Morales D., Ramirez G., Herrera-Arellano A., Tortoriello J., Zavala M., Zamilpa A. (2018). Identification of digestive enzyme inhibitors from *Ludwigia octovalvis* (Jacq.) P.H.Raven. *Evidence-based Complementary and Alternative Medicine*.

[18] Demilew W., Adinew G. M., Asrade S. (2018). Evaluation of the wound healing activity of the crude extract of leaves of *Acanthus polystachyus* delile (Acanthaceae). *Evidence-Based Complementary and Alternative Medicine: eCAM*.

[19] Suriyamoorthy S., Subramaniam K., Wahab F., Karthikeyan G. (2012). Evaluation of wound healing activity of *Acacia leucophloea* bark in rats. *Revista Brasileira de Farmacognosia*.

[20] Alsarayreh A. Z., Oran S. A., Shakhanbeh J. M. (2022). Efficacy of methanolic extracts of some medicinal plants on wound healing in diabetic rats. *Heliyon*.

[21] Kundu A., Ghosh A., Singh N. K. (2016). Wound healing activity of the ethanol root extract and polyphenolic rich fraction from *Potentilla fulgens*. *Pharmaceutical Biology*.

[22] Andreoli A., Ruf M.-T., Sopoh G. E., Schmid P., Pluschke G. (2014). Immunohistochemical monitoring of wound healing in antibiotic treated buruli ulcer patients. *PLoS Neglected Tropical Diseases*.

[23] Sari L. M., Suyatna F. D., Subita G. P., Auerkar E. I. (2016). Acute dermal toxicity study of *Areca catechu* Linn. extract in Sprague-Dawley rats. *Asian Journal of Pharmaceutical and Clinical Research*.

[24] Sari L. M., Suyatna F. D., Subita G. P., Auerkari E. I. (2014). Acute oral toxicity study of *Areca catechu* linn. aqueous extract in Sprague-Dawley rats. *Asian Journal of Pharmaceutical and Clinical Research*.

[25] Rekha R., Kashmira G., Poonam S., Surendra S. (2015). Development of wound healing herbal formulation herbal wound guard. *International Journal of Scientific and Research Publications*.

[26] Rostami H., Mohammadi R., Asri-Rezaei S., Tehrani A. A. (2018). Evaluation of application of chitosan/nano selenium biodegradable film on full thickness excisional wound healing in rats. *Iranian Journal of Veterinary Surgery*.

[27] Tottoli E. M., Dorati R., Genta I., Chiesa E., Pisani S., Conti B. (2020). Skin wound healing process and new emerging technologies for skin wound care and regeneration. *Pharmaceutics*.

[28] Tai Y., Woods E. L., Dally J. (2021). Myofibroblasts: function, formation, and scope of molecular therapies for skin fibrosis. *Biomolecules*.

[29] Broughton G., Janis J. E., Attinger C. E. (2006). The basic science of wound healing. *Plastic and Reconstructive Surgery*.

[30] Sharma A., Khanna S., Kaur G., Singh I. (2021). Medicinal plants and their components for wound healing applications. *Future Journal of Pharmaceutical Sciences*.

[31] Rajan D. S., Rajkumar M., Kumarappan C. T., Kumar K. L. S. (2013). Wound healing activity of an herbal ointment containing the leaf extract of *Ziziphus Mauritiana* Lam. *African Journal of Pharmacy and Pharmacology*.

[32] Suguna L., Singh S., Sivakumar P., Sampath P., Chandrakasan G. (2002). Influence of *Terminalia chebula* on dermal wound healing in rats. *Phytotherapy Research*.

[33] Azis H. A., Taher M., Ahmed A. S. (2017). In vitro and in vivo wound healing studies of methanolic fraction of *Centella asiatica* extract. *South African Journal of Botany*.

[34] Desjardins-Park H. E., Foster D. S., Longaker M. T. (2018). Fibroblasts and wound healing: an update. *Regenerative Medicine*.

[35] Schultz G. S., Chin G. A., Moldawer L., Diegelmann R. F. (2011). Principles of wound healing. *Mechanisms of Vascular Disease*.

[36] Kumar V., Delhi N., Khan A. A., Nagarajan K. (2013). Animal models for the evaluation of wound healing activity. *International Bulletin of Drug Research*.

[37] Orsted H. L., Keast D., Forest-Lalande L., Françoise M. (2015). Basic principles of wound healing. *Wound Care Canada*.

[38] Velnar T., Bailey T., Smrkolj V. (2009). The wound healing process: an overview of the cellular and molecular mechanisms. *Journal of International Medical Research*.

[39] Cañedo-Dorantes L., Cañedo-Ayala M. (2019). Skin acute wound healing: a comprehensive review. *International Journal of Inflammation*.

[40] Singer A. J., Clark R. A. F. (1999). Cutaneous wound healing. *New England Journal of Medicine*.

[41] Rouhollahi E., Moghadamtousi S. Z., Hajiaghaalipour F. (2015). *Curcuma purpurascens* BI. rhizome accelerates rat excisional wound healing: Involvement of Hsp70/Bax proteins, antioxidant defense, and angiogenesis activity. *Drug Design, Development and Therapy*.

[42] Houghton P. J., Hylands P. J., Mensah A. Y., Hensel A., Deters A. M. (2005). In vitro tests and ethnopharmacological investigations: wound healing as an example. *Journal of Ethnopharmacology*.

[43] Reduan Mohd F. H., Mohd R. S., Sayuti N. S. A. (2021). Evaluation of dermal toxicity study of ethanolic extract of *Morinda citrifolia* fruit in *Spraque Dawley* rats. *The Thai Journal of Veterinary Medicine*.

[44] Teshome K., Gebre-Mariam T., Asres K., Perry F., Engidawork E. (2008). Toxicity studies on dermal application of plant extract of *Plumbago zeylanica* used in Ethiopian traditional medicine. *Journal of Ethnopharmacology*.

[45] Nyigo V., Mdegela R., Mabiki F., Malebo H. (2015). Assessment of dermal irritation and acute toxicity potential of extracts from *Synadenium glaucescens* on healthy rabbits, wistar albino rats and albino mice. *European Journal of Medicinal Plants*.

